# Lycopene Alleviates Lipid Dysregulation, Oxidative Stress, and Hypercholesterolemia in Obese Rats Subjected to a High‐Fat Diet

**DOI:** 10.1002/fsn3.70549

**Published:** 2025-07-07

**Authors:** Sana Noreen, Somia Shehzadi, Chukwuebuka Egbuna, Patrick Maduabuchi Aja

**Affiliations:** ^1^ University Institute of Diet and Nutritional Sciences, The University of Lahore Lahore Pakistan; ^2^ University Institute of Medical Laboratory Technology, The University of Lahore Lahore Pakistan; ^3^ African Centre of Excellence in Public Health and Toxicological Research (ACE‐PUTOR) University of Port‐Harcourt Rivers Nigeria; ^4^ Department of Biochemistry, Faculty of Biomedical Sciences Kampala International University Bushenyi Western Uganda Uganda

**Keywords:** anti‐inflammation, antioxidant, high‐fat diet, lycopene, metabolic syndrome

## Abstract

Obesity, driven by high‐calorie diets, is a global health concern closely associated with chronic illnesses such as cardiovascular disease and liver dysfunction. This study investigated the therapeutic potential of lycopene, a potent antioxidant found in tomatoes, in mitigating hyperlipidemia‐induced metabolic disorders in male rats. Obese rats induced by a high‐fat diet (HFD) showed significant increases in body weight (284.12 ± 4.33 g) and serum levels of total cholesterol (TC: 255.21 ± 2.24 mg/dL), triglycerides (TG: 199.23 ± 6.12 mg/dL), LDL‐C (171.27 ± 4.84 mg/dL), and liver enzymes ALT (62.10 ± 3.80 U/L), AST (92.41 ± 1.48 U/L), and ALP (148.18 ± 0.55 U/L), alongside elevated inflammatory cytokines TNF‐α (1279 ± 10.81 pg/mL) and IL‐6 (859 ± 17.3 pg/mL). Treatment with lycopene (30 mg/kg b.w.) over 6 weeks significantly reduced body weight (237.94 ± 3.14 g), TC (180.85 ± 4.99 mg/dL), TG (137.13 ± 5.61 mg/dL), LDL‐C (46.11 ± 2.67 mg/dL), and liver enzyme levels (ALT: 30.34 ± 5.07 U/L, AST: 71.19 ± 2.12 U/L, ALP: 126.30 ± 5.15 U/L). It also improved HDL‐C (48.15 ± 2.35 mg/dL) and antioxidant markers such as SOD (40.62 ± 2.20 U/dL) and CAT (63.66 ± 4.73 U/g), whereas decreasing MDA (29.45 ± 2.33 mM/g), TNF‐α (587 ± 12.9 pg/mL), and IL‐6 (301 ± 16.7 pg/mL) followed by lycopene (25 mg/kg b.w.). Histological analysis showed a marked improvement in liver architecture, with reduced fat accumulation and inflammation. These findings suggest that lycopene supplementation effectively counteracts obesity‐induced dyslipidemia, oxidative stress, and liver inflammation, supporting its potential role as a dietary therapeutic agent for metabolic and hepatic disorders. Future recommendations include conducting long‐term clinical trials in humans to validate these findings, exploring optimal dosages for dietary lycopene supplementation, and investigating its molecular mechanisms of action.

## Graphical abstract

Lycopene has the potential to improve lipid profiles and boost antioxidant enzyme activity, thus demonstrating its therapeutic potential in the management of obesity.

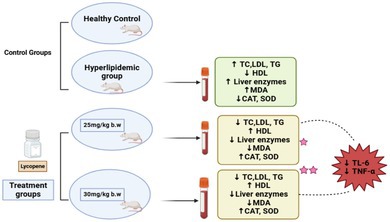

## 1 Introduction

Obesity affects 1.9 billion adults globally, with 650 million classified as obese. Dyslipidemia affects 60%–70% of obese individuals, leading to significant health consequences (Klein et al. 2022; Safaei et al. 2021). Cardiovascular diseases account for nearly 2.8 million deaths annually among obese populations. Type 2 diabetes, closely linked to obesity, affects about 90% of individuals with this metabolic disorder (Zhao 2021). Obesity increases the risk of endometrial, breast, and colon cancers and death by 20%–30%. Severe obesity can shorten life by 10 years. Nonalcoholic fatty liver disease (NAFLD) is also prevalent, affecting up to 90% of obese individuals (Al‐Daihan et al. 2018; Bays et al. 2021). Despite numerous public health initiatives, obesity rates have been increasing globally, with a threefold rise since 1975 (Nassir 2022). The etiology of obesity is complex, influenced by dietary patterns rich in high‐calorie, low‐nutrient foods, sedentary lifestyles, socioeconomic factors, genetic predispositions, such as FTO (fat mass and obesity‐associated gene) gene variants, and psychological factors including stress and depression. NAFLD is a prominent cause of chronic liver illnesses in the industrialized world, with a 25%–30% global prevalence (Bhat and Al‐daihan 2016; Klein et al. 2022). The assessment of NAFLD risk often involves monitoring liver function markers as well as lipid metabolism markers, including triglycerides and cholesterol levels. Adipose tissue, a key player in obesity‐related conditions, secretes bioactive peptides known as adipokines, which play significant roles in modulating inflammation and maintaining body weight homeostasis (Nassir 2022). The evaluation of oxidative stress biomarkers and antioxidants is critical in studying the redox state in NAFLD, as oxidative stress contributes to liver damage and disease progression. Dietary interventions, particularly those rich in plant‐based foods, have shown promise in combating obesity and related metabolic disorders (Powell‐Wiley et al. 2022).

Natural antioxidants have gained importance in human health due to their ability to treat various disorders and improve overall health (Martemucci et al. 2022). Poor intake of diet induces inflammation, ROS, and plasma lipid fluctuation, causing cardiovascular disease. Molecular oxidation must be restricted by antioxidants such as polyphenols, carotenoids, and ascorbic acid to avoid these diseases. Research has shown the effectiveness of these plants and herbs against oxidative stress. The pharmaceutical industry relies on natural products like microorganisms, marine organisms, and plants for their chemical diversity and drug‐like properties. Therapeutic plant medications are currently made using molecular, phytochemical, and biological approaches. Lycopene, a potent antioxidant found in fruits and vegetables, prevents oxidation and may be a food‐grade carotenoid source (Forman and Zhang 2021; Khan et al. 2021). It is a linear hydrocarbon with 11 conjugated double bonds that can undergo isomerization due to factors like temperature, light, and chemical reactions. Lycopene has 72 isoforms, with the most stable being 5‐cis lycopene. Isomerization affects its activity, with different physical and chemical properties (Caseiro et al. 2020). Humans cannot produce lycopene; the diet must provide it. Lycopene is an antioxidant present in tomatoes and other red fruits, and acts via several channels to prevent hyperlipidaemia and liver oxidative damage. It scavenges reactive oxygen species (ROS), therefore minimizing oxidative damage to lipids, proteins, and DNA and hence lowering oxidative stress indicators, including malondialdehyde (MDA) in liver tissues. Lycopene also increases the activity of antioxidant enzymes like catalase (CAT), glutathione peroxidase (GSH‐Px), and superoxide dismutase (SOD), hence reducing oxidative stress. Lycopene raises high‐density lipoprotein (HDL) levels while lowering total cholesterol, low‐density lipoprotein (LDL), and triglyceride levels, thereby modulating lipid metabolism. Moreover, it stimulates the Nrf2/HO‐1 signaling pathway, which raises antioxidant enzyme levels and hence lowers oxidative stress. Lycopene supplements have been demonstrated in non‐alcoholic fatty liver disease (NAFLD) models to increase mitochondrial activity, boost β‐oxidation, and lower liver lipid buildup. These interactions make lycopene a candidate for treatments in liver‐related diseases very interesting (Arballo et al. 2021). Obesity, driven by high‐fat diets (HFDs), leads to lipid dysregulation, oxidative stress, and inflammation, key contributors to metabolic disorders like hypercholesterolemia and fatty liver disease. Current treatments often carry adverse effects, highlighting the need for safer, natural alternatives. Lycopene, a powerful antioxidant found in tomatoes and red fruits, has shown potential in improving lipid metabolism, reducing oxidative stress, and lowering inflammatory markers. This study investigates lycopene's ability to mitigate lipid abnormalities, oxidative damage, and inflammation in obese rats, aiming to support its use as a dietary intervention for metabolic health.

## 2 Materials and Methods

Male Sprague–Dawley rats (180–200 g, 6–8 weeks old) were obtained from the University of Lahore's animal home in Pakistan. They were housed in a clean, ventilated environment (25°C–27°C) with a 12‐h light/dark cycle, divided into four groups of ten. The rats were fed on a consistent schedule and provided access to flowing tap water. They were acclimatized in a laboratory for 1 week before the experiment; a basal diet and water were provided ad libitum (Aly‐Aldin et al. 2015). The procedures employed were approved by the University of Lahore's Research Ethical Committee (IRB‐UOL‐FASH/826/2023), ensuring compliance with guidelines for animal care. The study followed humane guidelines to minimize animal suffering.

### 2.1 Standard Animal Diets

The basal diet was developed to nourish rats, as shown in Table 1, whereas the HFD, containing “cholesterol (4%) and cholic acid (1%),” was preserved at 4°C (Noreen et al. 2023). A Pakistani pharmaceutical firm supplied Basal and HFD.

**TABLE 1:** Basal and high‐fat diet composition (%).

Composition	Basal %	High‐fat diet %
Fat	5	5
Carbs	65	60
Protein	20	20
Fiber	5	5
Salt mixture	4	4
Vitamins	1	1
Cholic acid	0	1
Cholesterol	0	4

### 2.2 Collection of Lycopene

Lycopene supplement was collected from a local pharmacy, Lahore, Pakistan, and stored at room temperature for further use. The dose of the lycopene supplement was selected (Albrahim 2022).

### 2.3 Experimental Design

After a week of adaptation, rats (*n* = 40) were divided into two groups: a negative control (−ve control) and an HFD (+ve control). The HFD group was further divided into three groups for 6 weeks (Albrahim 2022) (Table 2). Hyperlipidemic groups were treated with lycopene (25 and 30 mg/kg b.w.) (Albrahim and Alonazi 2021; Elseweidy et al. 2022). Body weights (g) of rats from all groups were also recorded (Noreen et al. 2023).

**TABLE 2:** Experimental groups.

Groups		*n*	Control & Hyperlipidemic rats	Treatment	Duration
N (−ve)	N0	10	Healthy control	Basal diet	6 weeks
H (+ve)	H0	10	Hyperlipidemic rats	Basal diet	2 weeks on HFD 6 weeks on basal diet
	H1	10	Hyperlipidemic rats	Lycopene (25 mg/kg b.w.)	6 weeks
	H2	10	Hyperlipidemic rats	Lycopene (30 mg/kg b.w.)	6 weeks

### 2.4 Biochemical Examination

Ethylenediaminetetraacetic acid (EDTA) tubes were used to test blood samples for hematological parameters, and a chemical colorimetric method was used to measure lipid profiles. Blood samples were collected using anticoagulant tubes. At room temperature, the samples were allowed to coagulate for 30 min. To extract the serum, the samples were centrifuged for 10 min at 3000 rpm. Lipid profiles, including total cholesterol (TC), triglycerides (TG), high‐density lipoprotein cholesterol (HDL‐C), and low‐density lipoprotein cholesterol (LDL‐C), were assessed using the blood. Using commercially sold enzyme assay kits with a chemical colorimetric approach, the tests were carried out according to the manufacturer's standards. A UV–visible spectrophotometer helped compute the absorbance; the results were stated in mg/dL (Friedewald et al. 1972; Yahaya et al. 2024).

### 2.5 Liver Function Assay

Serum levels of aspartate aminotransferase (AST), alanine aminotransferase (ALT), and alkaline phosphatase (ALP) were assessed using commercially available diagnostic kits and enzymatic colorimetric techniques to evaluate liver function. Before centrifugation at 3000 rpm for 10 min to get clear serum, blood samples were first allowed to clot in tubes, and then used for analysis. AST and ALT activity was evaluated using a synthesis of pyruvate or oxaloacetate that interacts with 2,4‐dinitrophenylhydrazine (DNPH) to generate a colored hydrazone complex. ALP activity was evaluated using the hydrolysis of p‐nitrophenyl phosphate (pNPP) to p‐nitrophenol, which in alkaline conditions results in a yellow coloration. Each reaction mixture's absorbance at certain wavelengths was measured with a UV–Visible spectrophotometer; the enzyme activity was stated in units per liter (U/L) (Adeyemi et al. 2015).

### 2.6 Malondialdehyde Assay

The thiobarbituric acid reactive substances assay was used to quantify malondialdehyde (MDA) in the serum, therefore gauging lipid peroxidation. Reacting MDA with thiobarbituric acid (TBA) at high temperatures and in an acidic environment generates a pink MDA‐TBA complex serum samples were treated with TBA reagent, then heated in a boiling water bath for 15 min. The absorbance of the resulting solution at 532 nm upon cooling was measured using a UV–visible spectrophotometer. The MDA concentration was calculated using a standard curve and stated in millimoles per gram (mmol/g) of tissue (Ozdemir et al. 2009; Shafi and Chandrul 2017).

### 2.7 Assessment of CAT and SOD

Measuring the activity of catalase (CAT) and superoxide dismutase (SOD) allowed one to evaluate the antioxidant defense level of blood specimens. SOD's activity was tested using its capacity to stop superoxide radicals from autoxidizing pyrogallol or nitroblue tetrazolium. The absorbance was measured at 560 nm; SOD activity was expressed in units per deciliter (U/dL). Catalase activity was assessed using the rate of decomposition of hydrogen peroxide (H_2_O_2_). The absorbance at 240 nm was measured using a UV–Visible spectrophotometer after combining a known dosage of H_2_O_2_ with blood samples. The CAT activity was expressed using units per gram (U/g) of tissue (Samadi‐Noshahr et al. 2021).

### 2.8 Enzyme‐Linked Immunosorbent Assay (ELISA)

TNF‐α and IL‐6 levels were assessed using rat ELISA kits (R&D Systems, Minneapolis, USA). Using centrifugation, the serum of the treated and untreated groups was separated from the blood of all the rats; ELISA was then performed per kit guidelines. Every group underwent spectrophotometry using a microplate reader set at 450 nm (Lima et al. 2014).

### 2.9 Histopathology of Liver

Following the usual procedure, liver sections were fixed for a whole night in 10% formaldehyde buffer, then cut into 5 μm‐thick pieces and embedded in paraffin. The sections underwent routine histological investigation using hematoxylin and eosin (HE) staining. To assess the degree of hepatic damage or injury, the sections were next examined using an Olympus BX‐50 light microscope set to 400× magnification, and pictures were taken (Noreen et al. 2023).

### 2.10 Statistical Analysis

The study used a one‐way ANOVA test to analyze differences between control and treatment groups, with *p* values set at *p* < 0.05 (Steel and Torrie 1981).

## 3 Results and Discussion

Globally, obesity is attributed to the consumption of high‐calorie meals. There exists a strong correlation between obesity and certain chronic illnesses, underscoring the imperative of both prevention and treatment. Thus, this study studied obese rats' lycopene‐supplemented diets. Quantifying liver enzymes and rat lipids measures liver function. Additionally, inflammatory biomarkers were measured.

### 3.1 Effect of Lycopene on Body Weight

**FIGURE 1:**
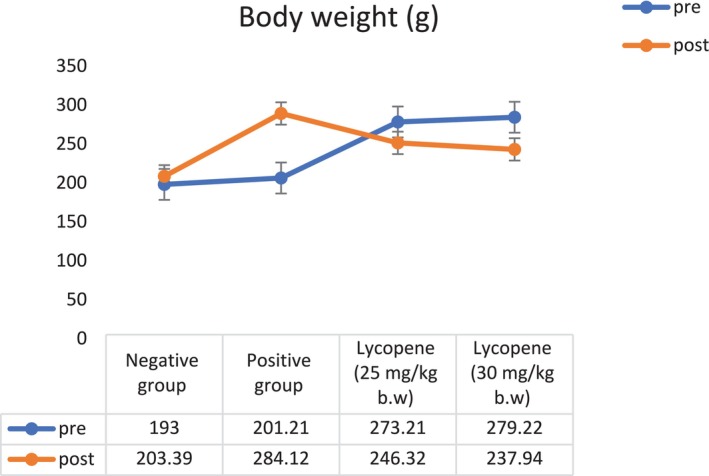
Effect of lycopene (25 and 30 mg/kg b.w.) on the body weight (g) among hyperlipidemic male rats.

Lycopene, a potent antioxidant found in tomatoes and other red fruits, has been extensively studied for its therapeutic potential in hyperlipidemia (Przybylska and Tokarczyk 2022). Its potent antioxidant qualities aid in lowering inflammation and oxidative stress, two factors that are increased in hyperlipidemic situations. It has also been demonstrated that lycopene enhances metabolic performance and aids in weight control. Furthermore, it may improve cardiovascular health by increasing high‐density lipoprotein (HDL) cholesterol and decreasing low‐density lipoprotein (LDL) cholesterol (Kwatra 2020). These benefits and their ability to stop fat accumulation suggest that lycopene might aid hyperlipidemic individuals in controlling their weight. Following 2 weeks of a HFD, the HFD group's mean body weights were considerably higher than those of the control group (193.00 ± 2.10 g), as were their Lycopene‐25 mg/kg (273.21 ± 2.03 g) and Lycopene‐30 mg/kg (279.22 ± 4.72 g). But after 6 weeks of lycopene supplementation, the lycopene‐30 mg/kg group experienced a significant (*p* ≤ 0.05) decrease in body weight (237.94 ± 3.14 g; a decrease of 41.28 g), followed by the lycopene‐25 mg/kg group (246.32 ± 3.10 g; a decrease of 26.89 g), as shown in Figure 1.

### 3.2 Effect of Lycopene on Lipid Profile

Tomatoes are a rich source of lycopene, a natural antioxidant known for its lipid‐lowering properties, including the reduction of low‐density lipoprotein (LDL) cholesterol, a major risk factor for atherosclerosis and cardiovascular diseases (Cheng et al. 2017). Lycopene also enhances high‐density lipoprotein (HDL) cholesterol levels, promotes cardiovascular health, and contributes to the reduction of total cholesterol (TC) and triglycerides (TG), making it a promising therapeutic agent for dyslipidemia (Müller et al. 2016). In the present study, as shown in Table 3, rats fed a HFD for 2 weeks exhibited a significant rise in serum TC (255.21 ± 2.24 mg/dL) and TG (199.23 ± 6.12 mg/dL) levels compared to the basal group (104.45 ± 1.22 and 149.78 ± 6.49 mg/dL, respectively). However, oral administration of lycopene at 25 and 30 mg/kg body weight per day for 6 weeks led to a marked reduction in TC (214.10 ± 2.72 mg/dL and 180.85 ± 4.99 mg/dL, respectively; *p* < 0.05) and TG levels. Furthermore, lycopene significantly lowered serum LDL‐C concentrations (67.41 ± 1.95 and 46.11 ± 2.67 mg/dL) and increased HDL‐C levels (36.27 ± 2.99 and 48.15 ± 2.35 mg/dL) compared to the untreated HFD group, demonstrating its beneficial role in improving lipid profiles.

**TABLE 3:** Impact of lycopene on the lipid profile (mg/dL) in male rats with hyperlipidemia.

Parameters	Week	Basal group	HFD group	Lycopene 25 mg/kg/day	Lycopene 30 mg/kg/day
TC	2nd	99.32 ± 6.13	255.21 ± 2.24	250.01 ± 1.19	250.28 ± 3.60
	6th	104.45 ± 1.22	256.11 ± 2.69^a^	214.10 ± 2.72^b^	180.85 ± 4.99^a^
TG	2nd	148.41 ± 3.96	199.23 ± 6.12	203.39 ± 3.93	212.83 ± 2.53
	6th	149.78 ± 6.49	200.23 ± 1.43^a^	153.12 ± 4.76^b^	137.13 ± 5.61^a^
LDL	2nd	30.21 ± 5.22	167.55 ± 2.28	169.93 ± 3.33	167.12 ± 1.58
	6th	32.23 ± 2.91	171.27 ± 4.84^a^	67.41 ± 1.95^b^	46.11 ± 2.67^a^
HDL	2nd	41.06 ± 2.30	26.67 ± 1.71	26.54 ± 4.25	25.03 ± 1.78
	6th	41.23 ± 1.93	29.27 ± 2.08^b^	36.27 ± 2.99^b^	48.15 ± 2.35^a^

*Note:* Data are mean ± SEM, *n* = 10.

^a^ Highly Significant level: *p* ≤ 0.05 compared to −ve control group.

^b^ Significant level: *p* ≤ 0.05 compared to a −ve control group.

### 3.3 Effect of Lycopene on Liver Function Test

Lycopene has shown potential to improve liver function in hyperlipidemic individuals by reducing oxidative stress, a key factor contributing to liver damage in lipid‐related disorders. Hyperlipidemia often leads to hepatic steatosis—excess fat accumulation in the liver—which can progress to liver injury (Albrahim 2022). As a potent antioxidant, lycopene neutralizes free radicals and mitigates oxidative stress, thus protecting hepatocytes from damage. This protective role is especially important in hyperlipidemic conditions characterized by elevated lipid peroxidation (Albrahim and Robert 2022). Research indicates that lycopene supplementation helps normalize liver enzyme levels by lowering serum concentrations of alanine aminotransferase (ALT) and aspartate aminotransferase (AST), both of which are biomarkers of liver dysfunction. Additionally, lycopene has been reported to reduce hepatic fat accumulation, thereby helping to prevent liver complications associated with hyperlipidemia (Elvira‐Torales et al. 2019). In the present study (Table 4), the positive control group showed significantly elevated levels of ALT (89.17 ± 3.22 U/L), AST (61.26 ± 2.19 U/L), and ALP (146.62 ± 2.16 U/L) after 2 weeks on a HFD, compared to the negative control group (44.10 ± 5.07, 17.20 ± 3.74, and 112.01 ± 1.67 U/L, respectively). However, oral supplementation of lycopene at 30 mg/kg for 6 weeks significantly (*p* ≤ 0.05) reduced these enzyme levels (ALT: 71.19 ± 2.12, AST: 30.34 ± 5.07, ALP: 126.30 ± 5.15 U/L), suggesting a protective effect against diet‐induced hepatic dysfunction.

**TABLE 4:** Effect of lycopene on liver enzymes (U/L) among hyperlipidemic male rats.

Parameters	Week	Basal group	HFD group	Lycopene 25 mg/kg/day	Lycopene 30 mg/kg/day
AST	2nd	44.10 ± 5.07	89.17 ± 3.22	91.42 ± 3.96	93.13 ± 3.12
	6th	47.34 ± 4.26	92.41 ± 1.48^a^	78.42 ± 4.38^b^	71.19 ± 2.12^a^
ALT	2nd	17.20 ± 3.74	61.26 ± 2.19	71.16 ± 2.64	69.02 ± 3.07
	6th	19.43 ± 2.42	62.10 ± 3.80^a^	53.21 ± 1.74^b^	30.34 ± 5.07^a^
ALP	2nd	112.01 ± 1.67	146.62 ± 2.16	159.23 ± 3.03	147.12 ± 4.81
	6th	113.52 ± 2.63	148.18 ± 0.55^a^	141.10 ± 3.45^a^	126.30 ± 5.15^a^

*Note:* Data are mean ± SEM, *n* = 10.

^a^ Highly Significant level: *p* ≤ 0.05 compared to −ve control group.

^b^ Significant level: *p* ≤ 0.05 compared to −ve control group.

### 3.4 Effect of Lycopene on the Activity of Catalase, SOD, and MDA Concentration

**FIGURE 2:**
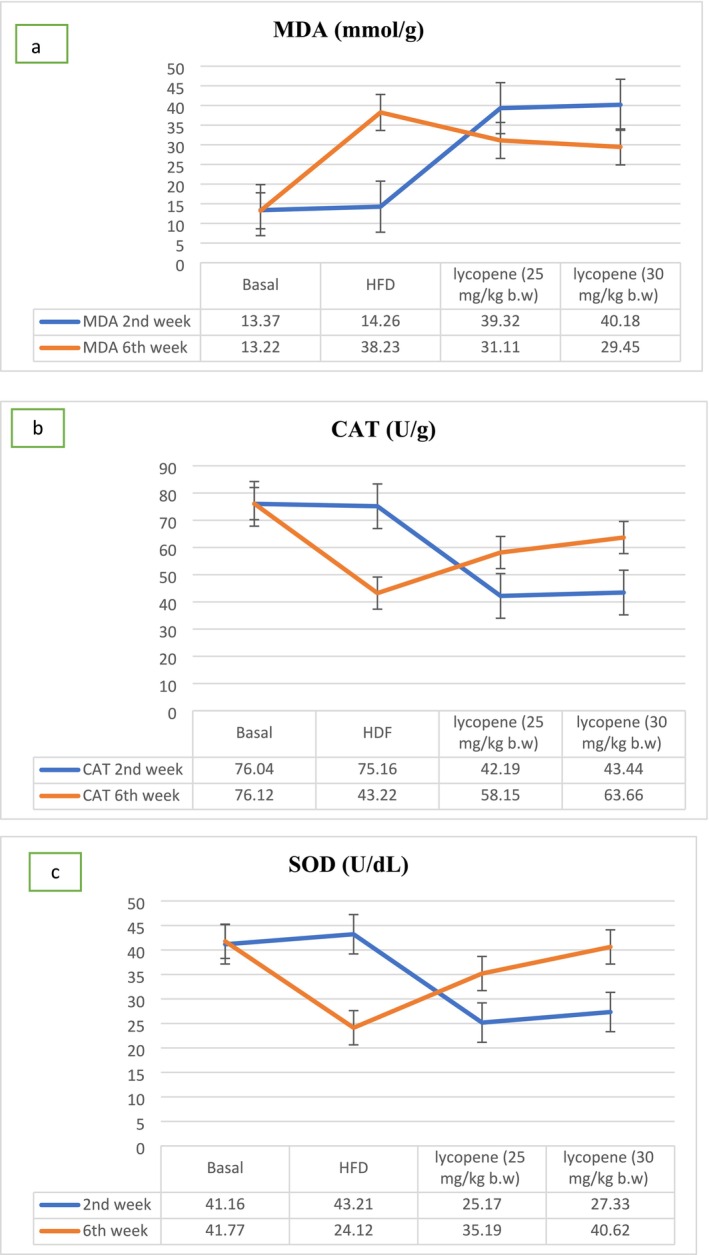
The effects of lycopene (25 and 30 mg/kg b.w.) on the MDA (a), SOD (b), and CAT (c) activities in the liver tissues of rats (*n* = 10).

Lycopene improves antioxidant enzymes and oxidative stress markers in hyperlipidemic individuals (Albrahim 2022). It enhances catalase activity, which reduces oxidative damage, and boosts serum superoxide dismutase (SOD) levels, which protect cells from oxidative damage (Salem 2015). Lycopene supplementation also decreases serum malondialdehyde (MDA) concentration, a lipid peroxidation marker, making it a valuable agent in combating oxidative stress and improving antioxidant enzyme activity (Mulkalwar et al. 2012). In comparison to −ve control rats, +ve control rats exhibited a significantly higher MDA level (38.23 ± 1.27 mM/g) and a marked decrease in CAT activity (43.22 ± 4.11 U/g), as shown in Figure 2 (13.22 ± 3.30 mM/g and 76.12 ± 2.28 U/g, respectively). Nevertheless, HFD rats that were given orally 25 and 30 mg/kg per day for 6 weeks had significantly lower blood MDA levels (31.11 ± 0.13, 29.45 ± 2.33 mM/g) and significantly higher activity of the CAT enzyme (58.15 ± 0.11, 63.66 ± 4.73 U/g, respectively) compared to the +ve control group. The results showed that when it came to the activity of SOD in rat serum, the +ve control group had a significantly lower level of activity (24.12 ± 1.22 U/dL) as compared to the −ve control group (41.77 ± 0.87 U/dL) (*p* ≤ 0.05). However, a considerably increased activity of the SOD enzyme (35.19 ± 1.45, 40.62 ± 2.20 U/dL, respectively) was noted in the lycopene‐supplemented group as compared to the +ve control group.

### 3.5 Effect of Lycopene on Anti‐Inflammatory Markers

**FIGURE 3:**
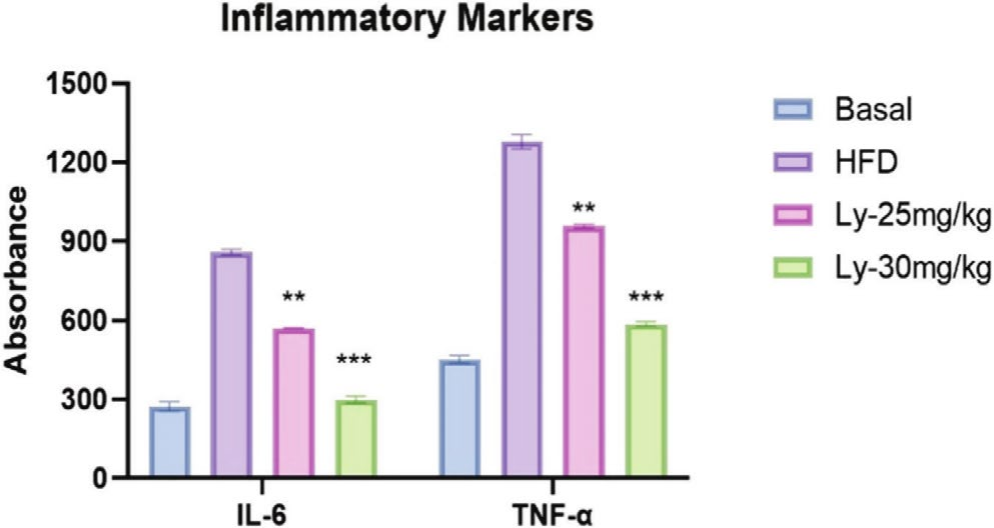
Analysis of inflammatory markers IL‐6 and TNF‐α in basal diet, HFD, and Ly‐25 and 30 mg/kg groups. Data are stated as the mean ± SEM of three replicas (*n* = 10 rats). **p < 0.001 indicates a significant change, whereas ***p < 0.0001 shows a very high significant change related to the HFD group.

Lycopene has anti‐inflammatory effects by reducing pro‐inflammatory markers like TNF‐α and IL‐6, which are linked to cardiovascular diseases (Przybylska and Tokarczyk 2022). By lowering TNF‐α levels, lycopene reduces chronic inflammation and associated risks, such as endothelial dysfunction and atherosclerosis (Albrahim 2022). This reduces IL‐6 levels, contributing to better cardiovascular health and reducing inflammation risks. The anti‐inflammatory impact of lycopene on HDF‐treated liver groups was validated by assessing TNF‐α and IL‐6 levels (Li et al. 2016). Both TNF‐α and IL‐6 presented a significant increase (*p* < 0.0001) in the HFD group: TNF‐α (1279 ± 10.81), IL‐6 (859 ± 17.3), compared to the group given the basal diet: TNF‐α (454 ± 6.53), IL‐6 (274 ± 7.08). However, a noteworthy decrease in both TNF‐α (587 ± 12.9) and IL‐6 (301 ± 16.7) (*p* < 0.0001) was seen in the Lycopene 30 mg/kg group when compared with the Basal diet group and HFD group, as shown in Figure 3.

### 3.6 Histopathology of Liver

**FIGURE 4:**
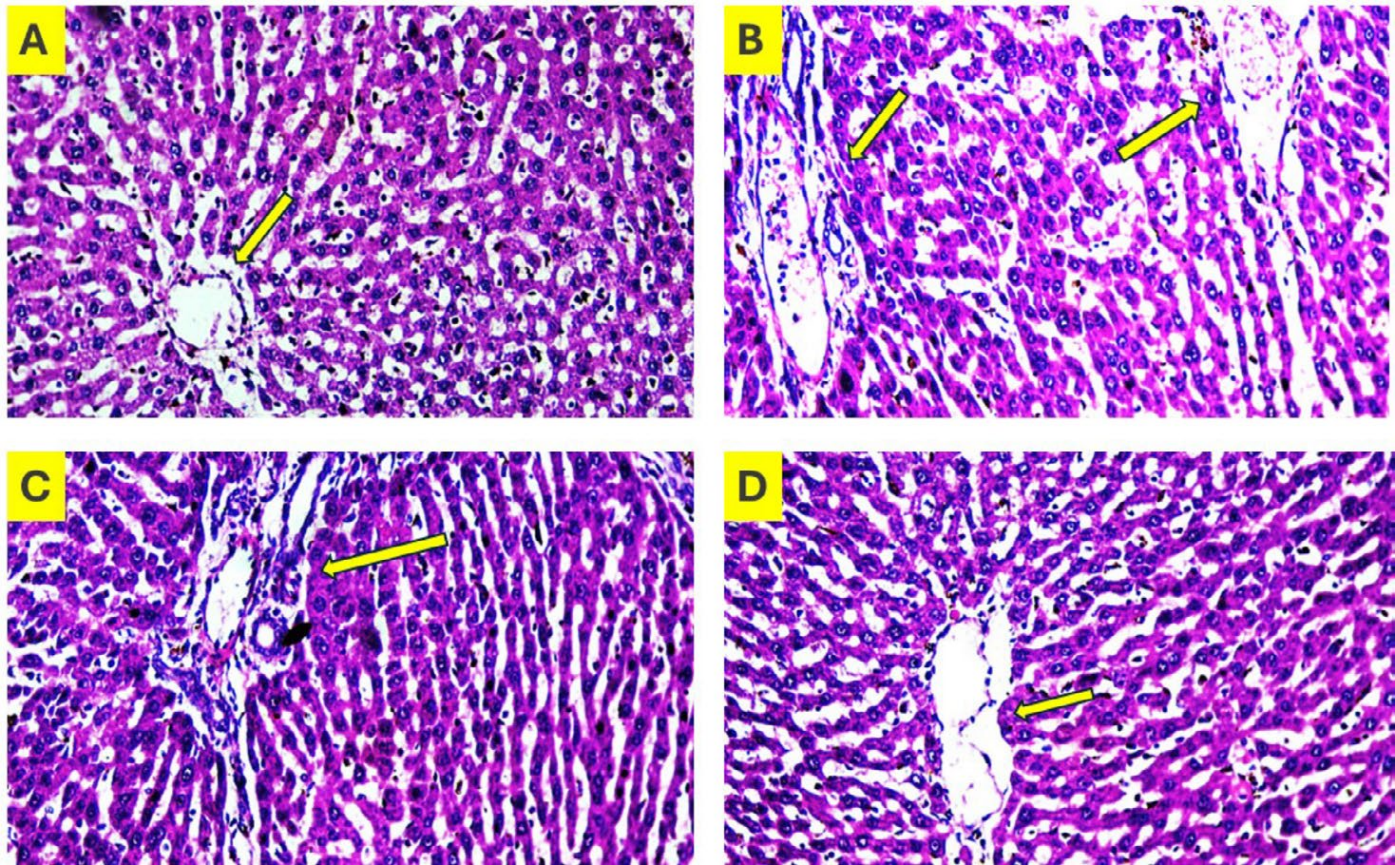
Histopathology analysis of rat liver sections: (A) Control group; (B) High‐fat diet group; (C) Lycopene treated with 25 mg/kg; (D) Lycopene treated with 30 mg/kg.

Lycopene improves liver health by reducing hepatic fat accumulation, reducing inflammation, enhancing liver cell integrity, and potentially preventing fibrosis (Sahin et al. 2018). Its antioxidant and anti‐inflammatory properties contribute to healthier liver tissue and function, making it a potential therapeutic benefit for individuals with liver‐related issues (Lee et al. 2019). Liver sections stained with HE of all groups were observed under a microscope. The control group displayed normal architecture of lobule; hepatocytes showed well defined nucleus and cytoplasm (Figure 4A). Whereas the injured group showed vacuoles filled with fat, inflammatory infiltration of cells, swelling, and degeneration in the central region of the lobules (Figure 4B). Furthermore, in the lycopene‐treated group with 25 mg/kg (Figure 4C), inflammation and degeneration were reduced; on the other hand, a significantly reduced inflammatory infiltration and fat droplet level were observed with lycopene 30 mg/kg (Figure 4D) compared with the injured and normal group.

## 4 Discussion

Hyperlipidemia ranks as a primary contributor to liver disease, cardiovascular disease, atherosclerosis, and premature mortality on a global scale (Hassen et al. 2022). It is imperative for worldwide public health to tackle obesity and its association with chronic diseases. Adequate nutrition has a crucial role in managing hyperlipidemia. Hyperlipidemia is defined as the presence of elevated concentrations of blood lipids and lipoproteins (Lonardo et al. 2013). Given the clinical importance of cholesterol as a lipid molecule in atherosclerosis, alterations in lipids and lipoproteins are highly adjustable risk factors for cardiovascular disease. The study consistently demonstrates an inverse relationship between HDL cholesterol and total body cholesterol. Hypercholesterolemia was linked to raised TC, LDL‐C, and VLDL‐C, and decreased HDL in rats fed a HFD (Noreen et al. 2023). The current study found that rats fed a HFD developed dyslipidemia in TG, total cholesterol, and LDL levels.

In obese hyperlipidemic rats, lycopene treatment at varying concentrations significantly reduced LDL levels and increased HDL, which is in line with a previous study that demonstrated lycopene's impact on lipid metabolism (Senkus et al. 2019). Our study also revealed that LYC co‐treatment significantly reduced LDL levels while raising HDL in obese rats. This finding is in line with earlier research showing that LYC significantly enhances blood lipid metabolism indicators (Salem et al. 2023; Senkus et al. 2019). According to earlier research, LYC can affect cholesterol metabolism in several ways. The primary mechanisms governing HDL levels in the blood have been revealed by the HDL metabolic pathway. HDL is made up of lipids, proteins, and free cholesterol and is esterified by Lecithin Cholesterol Acyltransferase (LCAT) and Cholesterol Ester Transfer Protein (CETP) (Alvi et al. 2017; Ossoli et al. 2016). Additionally, these two proteins regulate the conversion of VLDL, TG, and LDL to HDL cholesterol esters. It was examined how LYC affects HDL‐associated inflammation in moderately obese middle‐aged adults. They found that LYC administration reduced CETP and increased serum LCAT (Amaya‐Montoya et al. 2020; Bohn 2019). In contrast, LYC decreases cholesterol synthesis by inhibiting HMG‐CoA reductase and regulating LDL receptor and ACAT activity (Bays et al. 2021). Natural antioxidants may reduce free radical formation and oxidative damage, which can cause dyslipidemia and other HFD problems, according to recent research (Alagna et al. 2020; Albataineh et al. 2019; Ruiz‐Núñez et al. 2016).

This study examined the effect of lycopene supplementation on liver enzymes and lipid profiles of obese rats. Additionally, inflammatory indicators, oxidative stress, and antioxidant defense system responses were also assessed. Liver markers are necessary for many metabolic functions. Liver enzymes usually indicate inflammation or liver injury. Too high levels of ALT and AST are vital for the metabolism of amino acids. Raised ALP levels, which are linked to protein breakdown and bile acid metabolism, might point to liver disease or bile duct blockage. High levels of gamma‐glutamyl transferase, which aids in glutathione metabolism and detoxification, may point to liver disease or bile duct difficulties. Lactate dehydrogenase is found in many tissues, including the liver; it helps lactate be converted to pyruvate and, when raised, can signal liver disease and other organ problems (Cornelius 2020; Dietrich et al. 2016). Measuring liver function, diagnosing liver illnesses, and assessing the effectiveness of medication are all aided by monitoring these enzymes. AST, ALT, and ALP levels were higher in rats fed a HFD than in rats on a normal diet. The disintegration of hepatocyte membranes, which raises enzyme levels, may be the cause of this. However, in a rat model of non‐alcoholic fatty liver disease, serum AST and ALT levels in the HFD group with lycopene supplementation significantly decreased blood levels (Dietrich et al. 2016).

Immunological response and the development of disease both depend on inflammatory markers. TNF‐α, a pro‐inflammatory cytokine, attracts immune cells to areas of injury or infection and boosts them. Interleukin‐6 (IL‐6) controls the acute phase response by making the liver generate molecules like C‐reactive protein (CRP) (Ansar et al. 2016; Ehlting et al. 2021). Rising in reaction to systemic inflammation, an acute phase protein termed CRP monitors disease and responds to treatment. Upon release, IL‐1β induces the expression of interleukin‐8 (IL‐8), a potent chemokine that plays a crucial role in the inflammatory response. IL‐8 primarily functions to recruit and activate neutrophils, which are essential for combating infections and initiating tissue repair (Hart et al. 2020; Oikonomou et al. 2022). Finally, a non‐specific indication of inflammation, the Erythrocyte Sedimentation Rate (ESR), gauges the speed with which red blood cells sink in a test tube (Kumar et al. 2020). These indicators work together to identify, monitor, and treat a range of inflammatory and autoimmune illnesses by conveying the degree and severity of inflammation in the body. Throughout the trial, higher levels of the inflammatory cytokines TNF‐α and IL‐6 were also seen were higher levels of the inflammatory cytokines TNF‐α and IL‐6. The results of the present study fit those of past studies (Ansar et al. 2016). Previous research shows that LYC can have hepatoprotective and anti‐inflammatory benefits via reducing cytokine activity (Ibrahim et al. 2022). A LYC metabolite, apo‐10′‐lycopenoic acid, efficiently lowers hepatic inflammation in rats fed an HFD by suppressing TNF and IL‐6 and thus limiting cytokine generation (Bays et al. 2021; Ibrahim et al. 2022).

Though metabolic changes in oxidation, the Krebs cycle, and oxidative phosphorylation are raised, overnutrition generates too great ROS formation and oxidative stress in hepatic tissue (Dornas and Schuppan 2020). The current analysis revealed a significant increase in MDA in the HFD group. MDA and lipid peroxidation are elevated in obese patients, causing oxidative stress and cell membrane damage (Uçkan et al. 2022). The body needs MDA, CAT, and SOD to manage oxidative stress. Lipid peroxidation causes MDA, which indicates lipid damage. MDA levels over normal indicate oxidative stress and membrane damage. Hydrogen peroxide, a hazardous metabolic waste, is converted into water and oxygen by catalase (CAT), reducing oxidative damage. However, Superoxide Dismutase (SOD) converts highly reactive and harmful superoxide radicals into safer molecules like hydrogen peroxide, which catalase further breaks down. This chemical and enzyme combination reduces oxidative stress. CAT and SOD protect cells and tissues from oxidative stress by eliminating harmful reactive species, whereas MDA assesses oxidative damage (Panic et al. 2022).

Lycopene decreased obesity‐induced oxidative stress and boosted antioxidants. Lycopene's free radical‐scavenging antioxidant activities control SOD and CAT production (Panic et al. 2022). LYC suppresses singlet oxygen and free radicals better than β‐carotene and α‐tocopherol (Martemucci et al. 2022). Lycopene's chemical makeup and liposolubility may reduce oxidative stress (Müller et al. 2016). In a previous study, lycopene (5 mg/kg) reduced MDA levels in hyper‐homocysteine Wistar rats over 3 months, supporting our results that it controls redox imbalances (Salem 2015). SOD and CAT activity increased in NAFLD‐model Sprague Dawley rats following 4 weeks of lycopene (20 mg/kg) therapy (Li et al. 2016). Lycopene reduces MDA and increases CAT in a rat model of hepatic ischemia and reperfusion (Sahin et al. 2018). Lycopene is not an essential vitamin for health, but it can provide numerous major advantages that can help with general well‐being. Lycopene, a strong antioxidant found predominantly in tomatoes and other red fruits and vegetables, helps neutralize free radicals and decrease oxidative stress, which has been linked to a variety of chronic illnesses (Przybylska and Tokarczyk 2022). Its possible health advantages include lowering the risk of cardiovascular disease by improving lipid profiles and blood pressure, guarding against some forms of cancer, and promoting eye health by lowering the risk of age‐related macular degeneration (Salem 2015). Furthermore, lycopene's anti‐inflammatory effects can help regulate hyperlipidemia and promote liver function. Although lycopene‐rich foods are not required for survival, integrating them into a well‐balanced diet can help to maintain these health advantages and contribute to a better lifestyle.

## 5 Conclusion

In summary, this study shows that lycopene supplementation has positive effects on hyperlipidemic rats and has the potential to be used as a treatment for obesity and associated liver damage. By raising HDL cholesterol levels and decreasing total cholesterol, triglycerides, and LDL cholesterol, lycopene improved lipid profiles and dramatically decreased body weight. Additionally, as demonstrated by elevated catalase and superoxide dismutase activity and decreased malondialdehyde levels, lycopene improved antioxidant activity, lowering oxidative stress. The decrease of pro‐inflammatory markers TNF‐α and IL‐6 demonstrated its anti‐inflammatory qualities, reduced hepatic fat buildup, and enhanced liver tissue integrity, according to histopathological examination. According to these results, lycopene may be a useful dietary supplement for the treatment of liver diseases, obesity, and hyperlipidemia. Its possible therapeutic uses also call for more research in clinical settings. However, further research is required to analyze key genes involved in lipid metabolism to assess the regulatory effects of lycopene on fatty acid oxidation and lipogenesis pathways.

## Author Contributions

**Sana Noreen:** conceptualization (equal), investigation (equal), methodology (equal), supervision (equal). **Somia Shehzadi:** conceptualization (equal), investigation (equal), methodology (equal). **Chukwuebuka Egbuna:** conceptualization (equal), investigation (equal), methodology (equal), validation (equal). **Patrick Maduabuchi Aja:** conceptualization (equal), data curation (equal), investigation (equal), supervision (equal).

## Conflicts of Interest

The authors declare no conflicts of interest.

## Data Availability

The data that support the findings of this study are available from the corresponding author upon reasonable request.
